# Editorial: New progress in cancer biomarkers and therapy

**DOI:** 10.3389/fmolb.2024.1388872

**Published:** 2024-03-07

**Authors:** Guohui Sun, Chengwei He, Jianhua Wang

**Affiliations:** ^1^ Beijing Key Laboratory of Environmental and Viral Oncology, College of Chemistry and Life Science, Beijing University of Technology, Beijing, China; ^2^ State Key Laboratory of Quality Research in Chinese Medicine, Institute of Chinese Medical Sciences, University of Macau, Taipa, China; ^3^ Beijing Municipal Key Laboratory of Child Development and Nutriomics, Capital Institute of Pediatrics, Beijing, China

**Keywords:** cancer, biomarkers, bioinformatics, tumor prognosis, tumor microenvironment, tumor resistance, tumor therapy

As we are aware, cancer poses a significant threat to human health, with projections suggesting it may surpass cardiovascular disease as the primary cause of premature death in many countries in the coming years, as per the latest data from “Global Cancer Statistics 2020” (Sung et al., 2021). Despite that sounds pretty terrible, there’s optimism surrounding advancements in tumor biology that could potentially improve this scenario (Nakamura et al., 2021; Bai et al., 2023; Loi et al., 2023). Of notable importance are cancer biomarkers, which carry substantial clinical implications, aiding in early detection, monitoring treatment progress, and predicting cancer prognosis (Sarhadi and Armengol, 2022; Wang and Deng, 2023). Over the past several decades, analytical techniques in tumor biomarker research are essential tools for identifying, quantifying, and characterizing molecules or proteins that can serve as indicators of cancer presence, progression, or response to treatment. These techniques encompass a range of methods, each offering unique advantages and applications, including but not limited (Jayanthi et al., 2017; Japp et al., 2021; Eftekhari et al., 2022; Lee et al., 2023): immunohistochemistry (IHC), enzyme-linked immunosorbent assay (ELISA), flow cytometry, fluorescent probe, biosensor, polymerase chain reaction (PCR), next-generation sequencing (NGS), mass spectrometry (MS), surface enhanced raman spectroscopy (SERS), liquid biopsy, bioinformatics, *etc.* These analytical techniques play a crucial role in the discovery, validation, and clinical application of tumor biomarkers, contributing to improved cancer diagnosis, prognosis, and treatment outcomes. In this Research Topic, our focus lies on the theme of “cancer biomarkers,” encompassing various aspects such as: 1) the exploration of new cancer biomarkers, which includes novel analytical techniques, identification of new molecules or proteins, and their association with emerging cancer types; 2) understanding the biological properties inherent to cancer biomarkers; 3) elucidating the mechanisms of action and biological significance of these biomarkers; and 4) exploring how biomarkers can inform chemotherapy and biotherapy strategies. In addition to traditional methods, cutting-edge tools and concepts such as proteomics, bioinformatics, machine learning, artificial intelligence (AI), and single-cell sequencing play pivotal roles in the discovery and comprehension of cancer biomarkers. Particularly noteworthy is the potential of cancer biomarkers in shaping clinical approaches to cancer prevention and treatment, warranting in-depth investigation.

In this Research Topic, we successfully published 12 high-quality papers that will be of interest for researchers in cancer biomarkers. Of the 12 papers, seven are related to the cancer risk prediction, three are related to the potential of small metabolites as cancer biomarkers, the application of fluorescence *in situ* hybridization (FISH) in cancer diagnosis and the influencing factors of caner biomarkers. Another two papers are reviews that summarize the biological implications of potential cancer biomarkers.

The opening paper conducted by Yang et al. highlights the significance of M2 macrophage-related genes in both the treatment and prognosis of pancreatic cancer. Using multiple bioinformatic tools, the authors constructed the risk predictive models and revealed that the risk levels were closely associated with tumor mutational burden, immune checkpoint blockade related genes, and immune cells. Moreover, they predicted the potential associations between different risk models and the efficacy of chemotherapeutic agents (e.g., metformin, paclitaxel and lapatinib), and underscored the utility of WGCNA-based analysis of M2 macrophage-related genes in prognosticating outcomes for pancreatic cancer patients and suggested novel avenues for immunotherapy in this context. In the next work, Deng et al. explored the role of lysine acetylation-related genes (LARGs) in oral squamous cell carcinoma (OSCC) using bioinformatic methods. They developed a lysine acetylation-related prognostic model using TCGA OSCC datasets and revealed that patients with lower risk scores had better prognoses in both the overall cohort and within the subgroups, thus offering a new model for classifying OSCC and determining its prognosis. Xie et al. examined Centrosomal Protein 55 (CEP55) as a cancer-testis antigen, assessing its expression in tumors and its impact on prognosis. They developed a CEP55-based model for hepatocellular carcinoma (HCC), linking high CEP55 levels to increased cell cycle activity, proliferation, and immune pathways. CEP55 correlated with immune modulators and showed promise in predicting responses to immune checkpoint inhibitors (ICIs). The study also associated CEP55 expression with specific HCC molecular subtypes and devised a nomogram for survival prediction. Overall, CEP55 may serve as a prognostic biomarker and predictor of ICI efficacy, potentially influencing tumor immune microenvironments across various cancers. In head and neck squamous cell carcinoma (HNSCC), tumor microenvironment (TME) also plays an important role in tumor progression, however, the relationship between TME characteristics and the prognosis of HNSCC patients remains poorly understood. Wan et al. utilized the “estimate” R package to calculate the immune and stromal cell scores and identify seven new markers. They constructed a risk model categorizing HNSCC samples into low- and high-risk groups, validated for accuracy using Kaplan-Meier survival and ROC analyses. CIBERSORT algorithm revealed significant differences in immune cell infiltration between risk groups. These findings shed light on TME roles and unveil new prognostic biomarkers for HNSCC patients.

In acute myeloid leukemia (AML), ferroptosis offers potential against drug resistance. Using TCGA data, Wu et al. created a prognostic model incorporating eight prognosis-related ferroptosis genes (PRFGs) via LASSO regression. The constructed nomogram, integrating LASSO score, age, and cytogenetic risk, accurately predicts overall survival. Low-risk patients demonstrate significantly improved survival. Gene expression analyses reveal the relevance of PARPs with different clinical subgroups and the overall survival in AML patients. Immune-related pathways influence prognosis disparities, suggesting a TME role. Combining PARP inhibitors with ferroptosis inducers shows promise as an AML therapy. This comprehensive approach aids in patient stratification and prognosis, offering novel treatment avenues. In another paper, Geng et al. developed an extracellular matrix (ECM)-based prediction model for ovarian serous adenocarcinoma survival using AI techniques. Analyzing TCGA-OV data, 15 key ECM genes were identified, validating the ECM risk score’s predictive efficacy. Multivariate COX analysis revealed independent prognostic factors. High ECM risk score patients responded better to thyroglobulin-targeted immunotherapy, while low-risk patients benefitted from RYR2 gene-related treatment. Low-risk patients showed elevated immune checkpoint gene expression and immunophenoscore levels, indicating better immunotherapy response. The ECM risk score serves as a reliable tool for immunotherapy sensitivity assessment and ovarian cancer prognosis prediction. Zhao et al. reported an interesting study aimed to identifying clinical-significant circadian clock (CC)-related genes in ovarian cancer (OC). Using TCGA data, 12 CC genes was analyzed to generate a Circadian Clock Index (CCI). High CCI correlated with poor overall survival (OS) and immune biomarkers. WGCNA identified a CCI-correlated gene module, yielding 15 hub genes significantly associated with OS and immune cell infiltration. Upstream regulators such as transcription factors and miRNAs of key genes were also predicted. These findings reveal 15 crucial CC genes having indicative value for OC prognosis and immune microenvironment, offering insights into OC molecular mechanisms.

Small molecule metabolites may serve as ovarian cancer biomarkers, yet causal links are unclear. Utilizing Mendelian randomization, Chang et al. identified 242 single nucleotide polymorphisms (SNPs) correlated with small molecule metabolites as instrumental variables to elucidate the causal relationship. Six metabolites correlated with reduced ovarian cancer risk, including hexadecenoylcarnitine and methioninesulfoxide. Fifteen metabolites were associated with subtype cancers; methionine sulfoxide and tetradecanoyl carnitine linked to reduced risk in clear cell and high-grade serous cancers, while tryptophan elevated risk in endometrioid cancer. These findings suggest potential biomarkers for early detection and highlight metabolites’ etiological roles, warranting further investigation into underlying mechanisms. UroVysionTM FISH, often used for urothelial carcinoma (UC), may also detect carcinoma of non-urothelial lineages (CNUL). Ke et al. found that 64% of CNUL cases showed positive urine FISH results. Histological FISH results aligned with urine FISH in most cases, suggesting FISH’s applicability in CNUL diagnosis. Squamous cell carcinoma antigen (SCCA) is a specific biomarker of squamous cell carcinoma, however, the elevation of SCCA in pneumonia patients without malignancy has not been studied. Wang et al. analyzed the influencing factors of SCCA elevation in community-acquired pneumonia patients. Among 309 community-acquired pneumonia (CAP) patients with normal serum indicator levels, 46.3% showed elevated SCCA. Age inversely affects SCCA elevation (OR = 0.97), while higher body temperature significantly increases risk (OR = 3.75). Patients in higher quartiles of body temperature face substantially elevated SCCA risk. Thus, age and body temperature influence SCCA levels in CAP patients, with higher temperatures indicating heightened SCCA risk.

The last two papers are reviews by Yang et al. and Huang et al., respectively. Tyrosineprotein kinase-1 (ROS1) gene rearrangements occur in 0.9%–2.6% of non-small-cell lung cancers (NSCLCs). Targeting ROS1 can effectively inhibit tumor growth, offering clinical benefits. Yang et al. synthesizes insights into ROS1 rearrangements in NSCLCs, covering their oncogenic mechanisms, prevalence, detection methods, molecular features, therapeutic options, and drug resistance mechanisms. Abnormal translation regulation, crucial in cancer, involves eukaryotic translation initiation factor 4A1 (eIF4A1), an RNA helicase. It is regulated by microRNAs and long non-coding RNAs, impacting tumor cell proliferation and metastasis. Huang et al. summarized that eIF4A1 could serve as a biomarker for tumor diagnosis, staging, and outcome prediction, aiding precision medicine and targeted therapy. Small molecule inhibitors also show promise in clinical practice, supporting eIF4A1’s therapeutic potential.

In conclusion, we sincerely hope that the articles included in this Research Topic on cancer biomarkers will contribute to the research on cancer prevention and treatment, particularly by providing valuable insights into early diagnosis, biomarker-based therapy, and effective prognosis for tumors.
